# Symptomatic lumbar stenosis due to low-grade degenerative spondylolisthesis can effectively be treated with mere decompression

**DOI:** 10.1007/s00701-023-05667-7

**Published:** 2023-07-06

**Authors:** Judith M. P. van Grafhorst, Manon L. Dijkerman, Wilco C. Peul, Carmen L. A. Vleggeert-Lankamp

**Affiliations:** 1 University Neurosurgical Center Holland, LUMC, HMC, HAGA, Leiden, the Netherlands; 2 Department of Neurosurgery, Spaarne Gasthuis, Haarlem/Hoofddorp, Netherlands

**Keywords:** Lumbar stenosis, Degenerative spondylolisthesis, Decompression, Fusion, Reoperation rate

## Abstract

**Purpose:**

Adding instrumented spondylodesis to decompression in symptomatic spinal stenosis with degenerative spondylolisthesis is subject of debate. The presence of spondylolisthesis due to degeneration is an indicator of severe facet joint and intervertebral disc degeneration, and this may fit increased instability of the spine. We aim to establish the incidence of degenerative spondylolisthesis in spinal stenosis surgical candidates and to evaluate the incidence of failure of decompressive surgery without concomitant spondylodesis as initial treatment.

**Methods:**

Medical files of all operated patients for spinal stenosis between 2007 and 2013 were evaluated. Demographic characteristics, pre-operative radiological characteristics (level of stenosis, presence, and grade of spondylolisthesis), surgical technique, incidence, and indication for reoperation were summarised, as well as the type of reoperation. Patient satisfaction was classified as ‘satisfied’ or ‘unsatisfied’ after initial and secondary surgery. The follow-up was 6 to 12 years.

**Results:**

Nine hundred thirty-four patients were included, and 253 (27%) had a spondylolisthesis. Seventeen percent of the spondylolisthesis patients receiving decompression were reoperated versus 12% of the stenosis patients (*p*=.059). Reoperation in the spondylolisthesis group concerned instrumented spondylodesis in 38 versus 10% in the stenosis group. The satisfaction percentage was comparable in the stenosis and the spondylolisthesis group two months after surgery (80 vs. 74%). Of the 253 spondylolisthesis patients, 1% initially received instrumented spondylodesis and 6% in a second operation.

**Conclusion:**

Lumbar stenosis with and without (low-grade) degenerative spondylolisthesis can usually effectively be treated with mere decompression. Instrumented surgery in a second surgical procedure does not lead to less satisfaction with surgical outcomes.

## Introduction

Lumbar spinal stenosis may be due to degenerative spondylolisthesis, a condition in which one vertebra has slipped over the other due to facet joint degeneration and intervertebral disc degeneration [5, 12]. If the slippage leads to compression of the cauda equina and/or nerve roots, this can lead to neurogenic claudication and radiculopathy. Besides the typical symptoms in the legs upon walking and standing, patients may also suffer from back pain. This complex of symptoms results in a decreased quality of life wherein patients are limited in their physical functioning [13]. The mean age of patients with lumbar stenosis in combination with degenerative spondylolisthesis is around 70 years, and with the current ageing population, this problem is increasingly relevant [3].

It has been proven that surgical treatment is more effective than non-surgical treatment [17, 18]. Decompression is the generally accepted surgical technique for patients with lumbar stenosis. However, additional degenerative spondylolisthesis indicates severe degeneration of the facet joint and intervertebral disc, and this may fit increased instability of the spine [9]. Two studies have formed the basis for adding fusion to decompressive surgery in degenerative spondylolisthesis patients [1, 9]. Multiple studies have been performed to assess this matter, but conclusions differ [5, 6, 8, 16, 17, 19]. A recent systematic review concluded that there is no significant extra benefit from adding instrumented fusion to decompression in low-grade degenerative spondylolisthesis patients [4].

This retrospective study evaluates the results of surgical interventions in patients with stenosis, comparing those with and without degenerative spondylolisthesis. As a start, we want to elucidate the percentage of patients with spondylolisthesis in the patients with lumbar stenosis group. Secondly, the percentage of instrumented spondylodesis added to the decompressive surgical intervention will be evaluated. Thirdly, the reoperation rate in both stenosis patients and patients with stenosis due to spondylolisthesis and the type of reoperation (second decompression or instrumented spondylodesis) will be evaluated. Patient satisfaction will be retrieved from the surgeons’ notes, although this measure is subject to bias.

## Materials and methods

### Data collection

Data were retrieved from the medical files of all patients that underwent surgery for lumbar spinal stenosis in the non-university clinics, Spaarne Hospital (Hoofddorp) and Alrijne Hospital (Leiden), which are both parts of the Neurosurgical clinic of the Leiden University Medical Centre (LUMC, Leiden, the Netherlands). Data from spinal stenosis surgery patients performed between 2007 and 2013 were included, and data were collected up till 2019, ensuring that the follow-up period was 6 to 12 years.

### Inclusion criteria

Adult patients with symptomatic lumbar spinal stenosis with or without degenerative spondylolisthesis who were operated on for this indication were included. Symptomatic lumbar stenosis had to be characterised by neurogenic claudication. Stenosis could be due to spondylotic degeneration, ligamentous hypertrophy, discogenic protrusion, or a combination. The surgical technique had to be a decompression with or without concomitant instrumented fusion (posterior lumbar instrumented fusion with or without intervertebral fusion).

### Exclusion criteria

Patients who were previously operated on the lumbar spine were excluded. Patients with spondylolisthesis due to lysis and spondylolisthesis due to a traumatic event were also excluded. Patients with spinal deformities characterised by a Cobbs angle in the anteroposterior or lateral direction of more than 20 degrees (scoliosis) were excluded.

### Surgical procedures

#### Laminotomy

A small midline incision in the lower back is made after inducing general anaesthesia. The long back muscles are detached from the midline bone and lateralised. Decompression is applied via partial resection of the affected laminae, and no complete laminectomy is performed. This is why the procedure is called an interarcuate decompression. A flavectomy is performed to decompress the dural sac.

#### Instrumented spondylodesis

The same procedure as above is performed, but in order to get a good overview of the entry point of the screws for the instrumented fusion, the muscles have to be retracted substantially and then detached over a somewhat longer trajectory. The complete arch is removed, as well as the processus articularis inferior at both sides, to completely open the foraminal canal. If needed, the processus articularis superior of the inferior level is reduced. Discectomy is performed from both sides; if required, a re-alignment of the vertebrae can be accomplished. Under fluoroscopic guidance, pedicle screws are placed. Cages, filled with autologous bone, are introduced in the disc space from both sides. Rods are placed in the screws and affirmed to the screws.

### Outcome measurement

Data from medical files regarding the following items were extracted: demographic characteristics (age and gender), pre-operative radiological characteristics (level of stenosis, presence of spondylolisthesis, and grade of spondylolisthesis), and surgical technique.

For all patients that are operated on the lumbar spine, a postoperative consult is planned two months after surgery. This time point was chosen for postoperative clinical evaluation. For clinical evaluation, the notes written in the file by the neurosurgeon were evaluated. Patient satisfaction was extracted from the files (doctor’s notes) and defined as ‘satisfied’ or ‘non-satisfied’. Data on reoperation and outcome after reoperation were collected in the same manner as clinical evaluation after the initial surgery. Data on per- and postoperative complications were collected.

### Data analysis

Data is analysed with IBM SPSS 24.0. Patients are divided into two groups: A group of patients with lumbar stenosis without degenerative spondylolisthesis (the stenosis group) and a group of patients with lumbar stenosis with degenerative spondylolisthesis (the spondylolisthesis group). Patient demographic data, such as sex, stenosis level, and spondylolisthesis degree, is analysed by descriptive statistics. Continuous data were tested for normality with a Q-Q plot and are shown as mean values with standard deviation. The means are compared with an independent *t*-test. A significance level of 0.05 was maintained in comparing both groups. Categorical data is compared using chi-square tests and are demonstrated as numbers with/without percentages of the total, stratified by spondylolisthesis and stenosis. Clinical outcomes after initial surgery, reoperation rates, and secondary fusion rates were compared between the stenosis and the spondylolisthesis group. Finally, the patient satisfaction rates were compared after secondary decompression and secondary fusion.

## Results

### Demographics

A total of 934 patients were included, with 253 patients having lumbar stenosis combined with degenerative spondylolisthesis. Baseline characteristics are presented in Table 1. Patients in the spondylolisthesis group were slightly older compared with patients in the stenosis group. The mean age in the stenosis group is 77 years compared with a mean age of 79 in the spondylolisthesis group (*p*=.034; Table 1). Whereas male and female patients were almost equally represented in the stenosis group, female patients made up a larger share of the patients in the spondylolisthesis group (*p*=.000). In both groups, most patients suffered from stenosis at level L4-L5. Most spondylolisthesis patients suffered from a grade 1 spondylolisthesis (240 patients), whilst only nine patients had a grade 1–2 spondylolisthesis, and four patients suffered from a grade 2 spondylolisthesis.

**Table 1:** Patient characteristics of stenosis and spondylolisthesis patients

	Stenosis group (*n*=681)	Spondylolisthesis group (*n*=253)
Mean age (SD)*	77 (±10.7)	79 (±9.5)
Male % **	54.5%	30.4%
Level of stenosis (% per group)		
L1-L2 (%)	5 (0.7)	0 (0)
L2-L3 (%)	34 (5.0)	3 (1.2)
L3-L4 (%)	103 (15.1)	29 (11.5)
L4-L5 (%)	301 (44.2)	142 (56.1)
L5-S1 (%)	40 (5.9)	10 (4.0)
Multi-level (%)	198 (29.1)	69 (27.3)
Meyerding		
Grade 1	-	240
Grade 1/2	-	9
Grade 2	-	4

*Significant older patients in the spondylolisthesis group compared with the stenosis group (*p*=.034)

**Significant more female patients in spondylolisthesis group than in stenosis group (*p*=.000)

All but three patients initially received a decompression without concomitant fusion. The three patients receiving instrumented fusion as primary surgery all demonstrated a grade 1 spondylolisthesis.

### Clinical outcome after initial surgery

In the stenosis group, a satisfactory outcome two months postoperatively is reported in 79.6% of patients. This is comparable with a satisfaction rate of 74.3% two months postoperatively in the spondylolisthesis group (*p*=.059)

### Reoperation

**Fig. 1:**
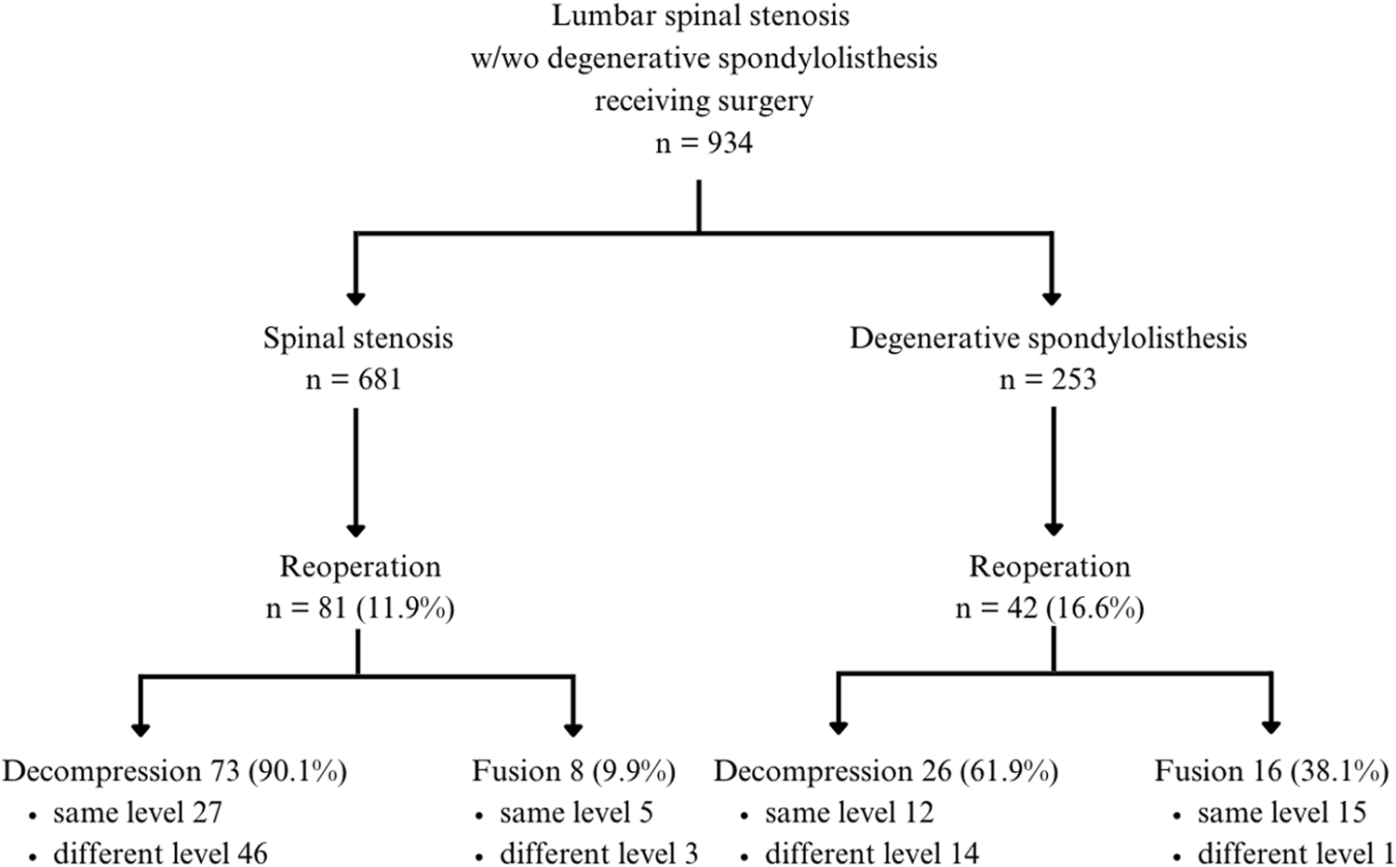
Type of reoperation

Reoperation was necessary for 81 of the 681 stenosis patients (11.9%), compared with 42 of the 253 spondylolisthesis patients (16.6%, *p*=.059). In the stenosis group, 73 (90.1%) reoperated patients received a second decompression; 46 were at a different level than the initial decompression, and 27 were at the same level as their initial decompression (Fig. 1). The other eight patients (9.9%) received an instrumented spondylodesis; 3 were at a different level than their initial decompression, and five were at the same level as their initial decompression (Table 2).

**Table 2:** Rates, type and level of reoperation in stenosis and spondylolisthesis group

	Stenosis group (*n*=681)	Spondylolisthesis group (*n*=253)	*p*-level
Reoperation (%)	81 (11.9)	42 (16.6)	.059
Secondary decompression (%)	73 (90.1)	26 (61.9)	.000
Different level	46	12	.134
Same level	27	14	
Secondary fusion (%)	8 (9.9)	16 (38.1)	
Different level	3	1	.053
Same level	5	15	

In the spondylolisthesis group, 26 (61.9%) reoperated patients received a second decompression; 12 at a different level than their initial decompression and 14 at the same level as their initial decompression. Sixteen patients in the spondylolisthesis group (38.1%) received a fusion in the follow-up of the initial decompression; 15 at the same level as their initial decompression. In total, 6.3% of the spondylolisthesis patients underwent instrumented spondylodesis versus 1.2% of the stenosis patients.

### Clinical outcome after secondary surgery

Two months after the second surgery, satisfaction rates were 67.1% in the stenosis group and 93.0% in the spondylolisthesis group (*p*=.005).

The overall satisfaction rate among patients who received a second decompression at another level than the initial decompression is 82.8% (stenosis and spondylolisthesis group; 80.4% and 91.7%, respectively, *p*=.359), whilst patients who received a second decompression at the same level as the initial decompression reported an overall satisfaction rate of 65.9% (stenosis and spondylolisthesis group; 48,1%% and 100%, respectively, *p*=.001).

In total, 24 patients received instrumented spondylodesis as a reoperation, and 83.3% had a satisfactory outcome two months after surgery. When divided into stenosis and spondylolisthesis patient groups, satisfaction rates were 62.5% and 93.8%, respectively (*p*=.127; Table 3).

**Table 3:** Patient satisfaction after initial operation and reoperation. If unsatisfied, divided into different level or same level reoperation

	Stenosis group (*n*=681)	Spondylolisthesis group (*n*=253)	*p*-level
Satisfaction after initial surgery (%)	542 (79.6)*	188 (74.3)	.059
Number of reoperated patients (%)	81 (11.9)	42 (16.6)	
Secondary decompression	73	26	
Clinical outcome:			
Satisfied (%)	50 (68.5)	25 (96.2)	.005
Unsatisfied (%)	23 (31.5)	1 (3.8)	
Unsatisfied in patients with different level reoperation (%)	9 out of 46 (19.6)	1 out of 12 (8.3)	.359
Unsatisfied in patients with same level reoperation (%)	14 out of 27 (51.9)	0 out of 14	.001
Secondary fusion	8	16	
Missing data on clinical outcome (%) *	1 (20)	0	
Clinical outcome:			
Satisfied (%)	5 (62.5)	15 (93.8)	.127
Unsatisfied (%)	2 (25)	1 (6.3)	
Unsatisfied patients within different level reoperation (%)	1 out of 3 (33)	0 out of 1	.505
Unsatisfied patients within same level reoperation (%)	1 out of 5 (20)	1 out of 15 (6.7)	.125

*Missing data; 6 cases of not clearly sated satisfactory outcome or not in stenosis group

**Missing data on the clinical outcome due to occurrence of cerebral infarct after surgery

## Discussion

Our results show that in patients with symptomatic lumbar spinal stenosis, almost one-third have a spondylolisthesis, the vast majority being low-grade. In the studied cohort, in which patients with an isthmic spondylolisthesis were excluded, only three spondylolisthesis patients were initially subjected to instrumented spondylodesis. Eventually, 6% of the spondylolisthesis patients were reoperated to receive instrumented spondylodesis.

In the spondylolisthesis group, 17% of patients were reoperated, and two-thirds were subjected to another decompression. Decompression on an adjacent level is likely to result from progressive degeneration in the lumbar spine and is considered part of routine spinal surgery of the lower back. However, a second decompression at the same lumbar level can be due to incomplete decompression in the first intervention. In degenerative spondylolisthesis, the upper arch of the target level is likely to imprint the dural sac, and incomplete removal may be the origin of persisting complaints. If the surgeon removed more of the lamina of the superior arch in the second surgical intervention, this led to a satisfaction rate of 96%, which is extraordinarily high in stenosis surgery. This could indicate that when performing decompression in spondylolisthesis patients, the surgeon has to increase the proportion of the upper arch of the stenotic level or even remove the whole arch to decompress the nerve roots fully.

Spondylodesis was added if the surgeon deemed it necessary to also remove the facet joint for adequate decompression. This resulted in a satisfaction rate of 94% after reoperation with spondylodesis.

Our regimen of performing mere decompression in spondylolisthesis patients with symptomatic stenosis can be concluded to be successful. The 17% reoperation rate in the spondylolisthesis group (10% decompression and 7% fusion surgery) was substantially lower compared to other studies [6, 8, 15]. The study of Brodke, with only 45–21 patients per study arm, demonstrated higher reoperation rates (24%) at 5-year follow-up after fusion surgery compared with decompressive surgery (8%) in patients with grade 1 degenerative spondylolisthesis and/or degenerative scoliosis [2]. Sato et al. reported a long-term reoperation rate of 33.8% after decompression and a reoperation rate of 14.4% after decompression and fusion surgery [15]. Forsth et al. reported reoperation rates of 21% after decompression and 22% after decompression and fusion surgery [6]. Ghogawala et al. showed a reoperation rate after decompressive surgery of 34% and 14% after decompression with fusion surgery [8]. It must be taken into account that the latter three studies were prospective. The patients were encouraged to return to the clinic to evaluate the surgical outcomes. As a result of this, one could argue that the threshold for a second surgery is lower.

In the stenosis group, 12% of patients were reoperated. Only 8 of these 81 patients received a spondylodesis, and only in five of these patients was the spondylodesis performed at the same level as the initial decompression. This intervention appeared to be successful, though, considering the high satisfaction percentage among these patients (80%; Table 3). The low number does not allow us to indicate a particular property of these patients that could predict the successful outcome of spondylodesis in these non-spondylolisthesis patients. The reoperation rate among the stenosis without spondylolisthesis patients in our studied cohort is somewhat lower than the reoperation rate found by Sajadi et al. (12% in our study vs. 14% (95% CI 13–16%) [14]. Sajadi et al. performed a systematic review assessing the reoperation rates among lumbar spinal stenosis patients without spondylolisthesis, with a follow-up period of at least five years. The difference between the included studies and our study is that our study either has a longer follow-up, a more homogenous population and -surgical method, or a much larger studied population. Therefore, our study contributes to the knowledge of lumbar spinal stenosis and spondylolisthesis patients needing decompressive surgery and the long-term reoperation rates.

Most of our stenosis patients who received a second decompression were reoperated on another stenotic level in the lumbar spine. As stated above, it is only rational to expect this to be the consequence of ongoing degeneration in the lumbar spine. It is thus only logical that this led to a satisfaction rate of 80%, comparable with the satisfaction rate after initial decompression. However, a second decompressive surgery at the same level as the initial one led to a patient satisfaction of only 50%. The absence of success may be explained by other factors in the complaints of leg- and back pain like vascular claudication, hip problems, arthrosis, or a combination of these.

Not only is adding instrumented fusion to decompression more and more used in spondylolisthesis patients, but an increasing trend is also seen in the USA in stenosis patients without spondylolisthesis [3]. The figures presented here demonstrate that there is no justification for that. In both patient groups, satisfaction after surgery was high, 74–80%. However, it has to be taken into account that the treating physician gave this qualification on satisfaction and is thereby prone to bias. Our study’s satisfaction rates are somewhat higher than other reported satisfaction rates in literature [10, 11, 14]. In Sajadi et al.’s systematic review, different satisfaction rates are reported, varying from 52 to 75%. It has to be considered that those satisfaction rates are gathered by various measurements. The randomised controlled trial by Försth et al. demonstrates patient-reported data in which only two third of the spondylolisthesis patients are satisfied after initial surgery regardless if they received concomitant instrumented fusion to their decompressive surgery [6]. However, just like us, outcomes of both decompression and fusion are comparable.

This study has several limitations. The most important limitation is that patient satisfaction was extracted from the notes of the treating neurosurgeon, who is biased for his surgical results. Besides, patient satisfaction remains a subjective outcome measure, which can vary with a patient’s overall quality of life. Another limitation is the variation in follow-up time, which may affect the occurrence of a second surgery. However, the majority of patients that are reoperated after lumbar spine surgery have this second intervention within two years [7], and the follow-up time that was ensured in the current study is more than two years. If patients had visited another clinic with persistent complaints, it would have been outside the notes available to us.

## Conclusion

Lumbar stenosis with and without (low-grade) degenerative spondylolisthesis can effectively be treated with sole decompression, leaving the majority of patients satisfied after the initial surgery. Most patients with degenerative spondylolisthesis can be treated with another decompression in case of persisting symptoms. Still, removing a significant part of the superior arch is advisable to avoid reoperation.
